# Towards energy sustainability: Exploring the nexus between global value chain participation and energy security in developing and developed countries

**DOI:** 10.1371/journal.pone.0296705

**Published:** 2024-01-23

**Authors:** Shengda Zhang, Shuang Lin, Chaofeng Wang, Pomi Shahbaz

**Affiliations:** 1 School of Economics and Management, Civil Aviation Flight University of China, Deyang, China; 2 School of Airport Engineering, Civil Aviation Flight University of China, Deyang, China; 3 Division of Management and Administrative Science, Department of Economics, University of Education, Lahore, Punjab, Pakistan; University of Kara, TOGO

## Abstract

International trade has a significant impact on global environmental quality and sustainable economic development. Global value chains (GVCs) have become a crucial component of international trade and development policy. The global production structure has become more complicated with the inclusion of domestic markets in GVC, putting significant pressure on world energy resources and environmental sustainability. Therefore, traditional trade measures no longer accurately reflect how global trade affects the energy security of developing and developed countries. Thus, this study is the first to use a panel-corrected standard error method to look at the relationship between GVC participation and energy security by using a global sample of 35 developed and 27 developing nations from 1995 to 2018. A feasible generalized least squares model was also applied to confirm the robustness of the model. Six indicators—foreign direct investment, industrialization level, capital formation, human capital index, political stability, and GVC—were used in this research to look at their impact on the four fundamental pillars of energy security (availability, applicability, sustainability, and affordability) for sustainable economic development. For developed countries, it was confirmed that there is a non-linear relationship between GVC participation and energy intensity, renewable energy consumption, and non-fossil fuel use. In the case of developing countries, the non-linear relationship in terms of all aspects of energy security was also confirmed. The findings also indicated that GVC’s involvement benefits all four dimensions of energy security in both developing and developed countries once it reaches a certain threshold. Our findings further support the impacts of long-term cointegration between GVC and energy security for sustainable economic development. Therefore, the nations must promote technology transfer and capacity building within GVCs for inclusive energy security. Similarly, they may foster sustainable practices through collaborative governance for a stable global energy network by acknowledging the positive impact of income levels on energy security.

## 1. Introduction

The world is facing the daunting challenge of fulfilling global energy requirements without contributing to climate change. Energy needs and climate change are two important policy issues that are linked and important to countries all over the world. Therefore, one of the most important and often-studied topics right now is how to make technologies that provide a steady and reliable source of energy. Energy use and climate change are only two of the many concerns of the researchers that threaten our ability to offer a steady and sustainable supply of energy. Rising economic activity and a growing population are primarily responsible for increasing energy demand worldwide. Global energy demand is expected to increase by 145% until 2030 and double by 2050 [1].

Worldwide primary energy consumption drives global economic growth, and primary energy consumption climbed from 155.22 EJ in 1965 to 556.63 EJ in 2020. By the end of 2020, the world will only have sufficient proved reserves of oil to last another 53.5 years, natural gas for another 48.8 years, and coal for another 139 years. More than 85 percent of total primary energy use comes from these three sources [2].

There has been a lot of inconsistency between countries in how they come up with and use new ideas and how they look for new energy sources. Emerging economies that are growing quickly are using and importing a lot more energy, while most developed economies have taken steps to use less conventional energy by using clean energy resources. For example, energy consumption in Europe decreased by 5% during the last two decades. Meanwhile, Japan reduced its energy use by about 15% during the same period. Energy consumption in the United States has been decoupled from GDP growth and has remained largely consistent, whereas Canada has reduced its energy use by almost 20% to lead an energy transition [3]. On the other hand, in Eastern Africa and India, primary energy consumption has more than doubled, and it has almost tripled in China and central Africa, leading to a 40% increase in global energy consumption. South Asian countries like Bangladesh, Afghanistan, India, Bhutan, the Maldives, Pakistan, Nepal, and Sri Lanka may have trouble growing their economies because of a lack of sufficient energy production in the region, which is the habitat of almost half the world’s population [4]. A large majority of the Southeast Asian population does not have access to clean energy sources and therefore they rely largely on traditional fuels like fuel wood and biomass for everyday activities like cooking and heating [5].

Trade patterns of industrialization over the last few decades have contributed to greenhouse gas emissions by putting pressure on the countries’ natural resources. However, the 2008–2010 oil crisis prompted concerns about energy security, underscoring the need to adopt clean energy sources that are less expensive, more reliable, easier to access, and less harmful to the environment compared to conventional energy sources. Greenhouse gases, which are emitted by traditional energy sources, are one of the main causes of climate change worldwide. Climate change poses a serious threat to global energy security and environmental sustainability. So, energy must be produced, delivered, and used in a way that does not deteriorate the environment [6]. This is also an absolute necessity for sustainable world economic development and the existence of humans on the earthly planet.

Energy security and trade patterns will play a critical role in propelling the economy towards sustained development. Almost one fifth of the world’s energy is used in the production of goods that are traded internationally [7]. Global value chains (GVCs), which are defined by "production deconstruction on a global scale" and "global trade integration," have changed since the 1980s because of the fast growth of communication and information technology [8]. The term "global value chain (GVC)" refers to a group of procedures and institutional structures that work together to create and share economic gain. GVC is a process that creates and provides value. A wide range of upstream and downstream actors from various nations as well as their efforts and resources, have an impact on the GVC outcomes. Global value chains (GVCs), which are typically driven by big multinational companies, have a great effect on the ecosystems of both developed and developing countries [9, 10]. Production has become a process that takes place in more than one country and in more than one factory due to specialization and global division. As a result, the production process of goods is hosted by many economies. Therefore, it is possible that a product produced in one nation using domestic or foreign energy will be transported to another nation for further processing before being used in a third nation [7].

In the modern world, energy is a key input that is needed for sustainable development. A key part of energy security is making sure that reliable and easily accessible energy resources are always available at a reasonable price [11]. Energy policy is a broad term, so its meaning often depends on the situation and the person’s judgment doing the analysis. Energy security is important for sustainable development because energy is such an important part of sustainable development. As a result of all of these factors, getting access to energy is often considered a critical component of any government policy. Borok et al. [12] described that a country’s economy moves forward sustainably when it has a variety of cheap and easy-to-use energy sources that can be used in large quantities. The recent global energy crisis has made it necessary to reevaluate creative approaches to guarantee the security of energy in a more sustainable way. Therefore, the current study is planned to assess the relationship between the participation of developed and developing countries in GVC and their energy security.

Energy security and GVC involvement have been studied globally under various circumstances. The current analysis included only a handful by distinguishing those connected to energy security from those focused on GVC participation. From 2000 to 2018, Lee and Wang [13] studied the energy security in 30 Chinese provinces. According to their findings, financial development and technological innovation improved energy security, although coastal regions and low-capital areas showed significant differences. Gong [14] investigated China’s worldwide energy expansion from a financial perspective, emphasizing precise strategic investment criteria and energy law discourse that addresses China’s foreign energy security. Reforming the energy industry and recognizing geopolitics in international energy investment arbitration are encouraged. Hu et al. [15] categorized 102 countries’ energy security into five categories, showing the "J" trend associated with the world geopolitical order. Developed economies benefit from efficient energy use, while developing countries lag due to a lack of innovation and low disposable income. Berdysheva and Ikonnikova [3] described how the global energy environment is transformed by new resources, technology, and climate commitment. Some economies have become energy independent, while others import more energy. A modified energy security index was used to analyze worldwide energy flows, including unconventional resources, EU renewables, and China’s gas use. Sharma [16] suggested a decentralized strategy throughout Asia and the Pacific using a 4 A framework of energy security (accessibility, affordability, availability, and acceptability). Local empowerment, indigenous clean energy technologies, Gandhian ideas for sustainable solutions, and less dependence on Western economies were emphasized. Torres [17] proposed a method to assess energy technology dependence in energy security assessments, and proposed a methodology to identify and assess such risks. This study used energy security, GVC, and innovation studies to develop a conceptual framework with three dependent dimensions: product supply, tacit knowledge, and codified knowledge. These factors will lower global GDP, especially in China and the US. Moreover, Ha [18], Jabeen [19], and Ali et al. [20], have focused on individual energy security indicators in the context of other variables like FDI, human capital, and political stability. Additionally, there are many more studies, e.g., Ali and Gniniguè [21] and Ali et al. [22], that have focused on the GVCs participation and its role toward the protection of the environment, but they lack the focus on energy security.

While existing studies have examined the relationship between energy security and engagement in the global value chain (GVC), there is a discernible research gap in terms of a comprehensive analysis of these dynamics in the context of both developed and developing nations. The current study has predominantly focused on specific regions or individual aspects of energy security, innovation, and global value chains (GVCs), which are frequently isolated from one another. This approach has resulted in a gap in understanding how these critical factors interconnect in developed and developing nations, with a specific emphasis on the contributions of human capital, foreign direct investment (FDI), and political stability. The aforementioned studies [3, 13–22], have provided valuable contributions in the fields of energy security, innovation, and renewable energy. However, they have not comprehensively explored the complex relationship between energy security and GVC involvement by considering human capital, political stability, and FDI, specifically across a set of developed and developing countries. Furthermore, although some studies have examined energy security and GVCs in certain regions or countries, a comprehensive analysis is required to uncover the complex interactions and variations within and between developed and developing nations. This research gap signifies the necessity of adopting a holistic and integrated approach to examine how these factors collectively influence energy security across developed and developing nations. This study has the potential to provide valuable insights for enhancing effective policymaking and strategic decision making for both governments and multinational enterprises. Therefore, this study seeks to address this critical gap by comprehensively investigating the interconnected factors of energy security, GVC involvement, human capital, political stability, and FDI across a wide panel of developed and developing countries. This study aims to make a valuable contribution towards enhancing a comprehensive understanding of the complex relationships at play, offering valuable insights that have practical implications for policymakers, businesses, and research scholars in the field of global economics and sustainable development. The remaining article is arranged as follows: The upcoming section explains the review of literature and hypothesis development for the study. The third section describes the material and methods, followed by the result section. The fifth section describes the discussion, and at last the conclusion is included in the sixth section.

## 2. Review of literature

The GVCs are a network of interconnected production, consumption, and distribution activities that involve different actors from different countries. GVCs is a network of scattered yet interconnected organizations that create value. Firms participating in GVC tend to specialize in one or a few products or services, and their key goal is to unite consumers, corporations, and governments on a worldwide scale. The GVC network’s advantages consist of its ability to assess the entire economic process, from producing a certain final product through utilization, including the larger size of such operations.

Even though developing countries make money when they join a value chain and sell their goods on the global market, Arce et al. [23] asserted that they risk hurting their own ecosystems in the process. According to the BP World Energy Statistics Yearbook, China used 71% of the world’s energy in 2012. Developing nations were mainly responsible for the global primary energy consumption’s net increase. Moreover, international trade contributes to almost one third of all pollution on the globe today [24]. Polluting industries in developed countries that face higher operational costs as a result of environmental legislation may relocate to developing countries in quest of more leeway for economic growth [25]. Several growing economies are combating this tendency by enacting stronger environmental and energy consumption rules. The developed world imports significantly more polluting items from the developing world than it emits as a result of its production [23].

Developing countries are improving their technology and enhancing their energy security in order to compete on the global market. Technological advancements that increase energy efficiency can lessen industrial energy intensity and pollution [26], which ultimately enhance the energy security. Increased international trade may have little effect on pollution levels in the industrialized world. Partners at GVC are being encouraged to think about the environmental impact of their products beyond the processes over which they have control. This is because people are becoming more aware of the harm that GVC activities do to the environment [27]. Stakeholders in GVCs take advantage of the opportunities and resources provided by their participation to improve environmental sustainability and reduce environmental impacts from operational activities along the value chain [27]. As a result of GVC’s advances, the new idea of environmental upgrading has arisen. Thus, GVC’s presence in both developing and developed countries has an effect on their energy security systems as well. GVC participation causes more natural resources to be used, which speeds up the use of scarce natural resources and makes the air quality worse [28]. This shows that GVC may have a negative impact on the energy security of participating countries, but after reaching a threshold level of participation, it may help improve energy security.

It is hard to implement and measure the GVC’s effect on the environment because of the technological and institutional transfer of environmentally friendly technologies and innovations among GVC partners [29], the complex nature of supply networks [30], and the technologically intensive nature of operations, which require strict regulation of their surrounding environment [29]. Due to these reasons, GVC’s benefits, public institutions, and administrative capacity to help sustain the environment and improve the energy security are undermined by its size and complexity.

As investment and technology move around the world, more ways have opened up for countries to improve their energy security and join the global economy. Participation in GVCs, on the other hand, will be very different between countries because of their different innovations and economic systems at different stages of growth and development [31]. More developed countries tend to be at the top of global industrial networks. This is because product standards and technical limits mean that industrialized countries export basic intermediate products with high added value and high energy security. Nevertheless, wealthy countries’ domestic energy consumption (an indicator of energy security) is decreasing [32] as a result of the shift of the manufacturing of energy- and pollution-intensive commodities to comparatively poor regions. In general, industrialized countries’ energy intensity is decreasing as they integrate into GVCs, particularly in sectors where outsourcing is frequent. When developing countries first enter GVCs, they concentrate on the bottom of the value chain, where they may benefit from low labor costs and abundant cheap resources [33]. Developing countries increased their energy use and pollution by taking over the manufacturing chains that developed countries had outsourced [34]. It’s important to note that participation in GVCs comes with both long-term benefits and short-term liabilities associated with the environment in the context of energy security. Joining GVCs has clear benefits, but because more natural resources are used, environmental damage and resource depletion are made worse. Sun et al. [10] also depicted that the use of production and assembly processes that use a lot of energy and materials by GVC actors hurts the environment. Therefore, we hypothesized the following hypothesis.

H1: GVCs participation of developing and developed countries may have negative impact on the energy security.

Environmental concerns are deeply related to energy security. The literature emphasizes that GVC participation can influence a country’s environmental policy and management of energy resources. Countries’ GVC membership automatically qualifies them to implement green practices and technologies [9]. Similarly, when GVCs are involved, countries are better able to use their own strengths to fight environmental damage [35] or advocate for green initiatives such as ISO 14001 certification and other pollution prevention measures [36]. As GVCs grow to include more and more high-tech and knowledge-intensive industrial links, developing nations have begun to learn from these technologies and use them, which strengthens their position in the global economy. A phenomenon called "low-end lock-in, which involves eliminating low-end value chains, may make it harder for some sectors to cut down on energy use [37], which makes it harder for trade to improve energy efficiency in developing countries. On the other hand, production methods in emerging countries have gotten better as new technologies and stricter rules for protecting the environment have been introduced. This has led to a steady drop in the amount of energy used per unit of output.

Wang et al. [38] have found an inverted U-shaped relationship between GVC participation and environmental degradation. They described the coexistence of two conflicting forces that determine the impact of participation in GVCs on the environment. First, participation in GVCs promotes economic growth by requiring more energy consumption, which has a greater negative impact on the environment and increases CO2 emissions per person. Second, when a country participates in GVCs, it may reap the rewards of the competitive effect and the technique spillover effect, both of which contribute to improving the environment. They concluded that it is possible that during the initial phase of engaging in global value chains (GVCs), the dominant factor may be scale or growth impact. However, as time progresses, the method spillover and competition effects become more prominent, resulting in an inverted U-shaped connection.

The energy required at each stage of the GVC production process varies. In contrast to the lower energy demands of downstream processes such as distribution and marketing, upstream processes such as manufacturing and assembly can have significant energy requirements [39]. As different GVC activities have varying energy needs, the relationship between GVC engagement and energy security is not always linear. Similarly, the degree to which a country is involved in a GVC can affect its priorities in terms of energy efficiency and technological innovation [31]. Increased participation in global value chains (GVCs), especially in high-tech industries, can encourage the development of new energy-efficient innovations [40]. This innovation can improve energy security by decreasing energy consumption and dependence on traditional energy sources, leading to a nonlinear improvement in energy security.

A potential non-linear relationship may arise when countries pursue economic growth through GVCs, with the need to adopt sustainable energy strategies to mitigate environmental risks. Moreover, GVC is critical for a country’s economic growth, and productivity. Knowledge-based economic growth compels countries to abandon traditional nonrenewable energy sources in favor of cutting-edge renewables [41]. As a result of technological improvements, countries may now use more energy-efficient machinery or produce more technically advanced items [42]. Sharing information and transferring cutting-edge technologies helps countries meet environmental requirements while also encouraging innovation and commercial growth [43]. With increased awareness, consumers are becoming more conscious of environmental norms and renewable energy sources. Notably, the growing demand for eco-friendly technology and commodities motivates GVC participants to use eco-friendly and energy-efficient technology for sustainable production. The firms need to improve their reputation and worth within the GVC [43] by concentrating on reducing negative effects on the environment or meeting environmental standards.

There might not be a linear relationship between energy security and GVC because GVC engagement can affect different aspects of energy security in both positive and negative ways. The positive effects of GVC on economic growth and energy security can only be realized when a participating country is in a better position. Rigo [44] reported that countries with more knowledge and new technologies are more likely to share them with the poorer countries. So, only nations that are more advanced within GVC will reap the aforementioned benefits of membership in terms of ensuring the stability of their energy systems and the sustainability of their environmental practices. Based on the above discussion, this study hypothesized a nonlinear relationship between GVC and energy security.

H2: There exists a non-linear relationship between GVC’s participation of developing and developed countries and energy security systems

## 3. Materials and methods

### 3.1. Energy security and its dimensions

Perspectives on what constitutes energy security vary widely across individuals, organizations, nations, geographical regions, and historical epochs. Scientists and engineers often talk about energy security, and they often list energy R&D, innovation, and tech transfer platforms as important factors of energy security. According to the World Bank, the important pillars of energy security are supply diversification, energy efficiency, and price stability [45]. Energy security, in the eyes of advocates and end users, is the availability of reliable energy services at an affordable price.

The idea of energy security as we know it today came about in the early 1800s, when coal was used more and more to power battleships and other military equipment. Global concerns about energy security came to the fore during the two world wars, the energy crisis of the 1970s, and the two Iraq invasions. Depending on the administration in power, achieving energy security in the United States of America has meant doing everything from halting all oil imports to restricting imports to only countries in the Middle East. It has also meant gradually weaning the nation off oil [46]. The concept of nuclear nonproliferation was added to the field of energy security after the 1970s nuclear power boom.

But none of this helps figure out the best institution, government, or short-term energy security plan. Defining energy security is challenging because it means different things to different countries and has changed over time. This makes it hard to say what kind of energy security is best or most beneficial. Sovacool and Brown [47] came up with the idea of energy security. They described that it "should take into account the interrelated issues of availability, efficiency, affordability, and ecological responsibility." In response to the ’energy security paradigm shift,’ the Asia-Pacific Energy Research Centre proposed a new definition of energy security,’ emphasizing the four A’s: availability, accessibility, affordability, and acceptability [48]. The framework gives a whole picture of the energy condition of developing and developed countries by utilizing four indicators for each of the four A’s (availability of resources, applicability of technology, acceptability by society, and affordability of energy resources).

The goal of the 4-As approach to assessing energy security is to do so along four dimensions, such as availability, applicability, acceptability, and affordability. We used the six variables that cover the four A’s of energy security to investigate how GVC affects energy security. International energy statistics from the United States Energy Information Administration were used to compile statistics for energy security. The availability is shown as a percentage of the ratio between the amount of primary energy produced and the amount of primary energy used, and the non-fossil consumption is measured by taking into account the proportion of non-fossil energy consumption in total energy consumption.

The applicability statistic takes into account a region’s ability to utilize and develop its own domestic energy sources. If new technology could be used to decrease energy waste and increase energy conservation, it would be a great illustration of increased performance on the applicability scale, allowing for longer durations of use with the same energy reserves. In a similar vein, as a nation embraces new technologies that increase its domestic energy reserves, its applicability increases (by extracting previously inaccessible resources). For this, we consider the intensity level of primary energy in countries. A decrease in energy intensity may indicate improved energy efficiency and conservation, leading to greater energy reliability. A shift in the industry’s underlying structure may also account for the observed decline in energy consumption. For instance, investment may be shifting away from sectors where returns are low and towards those where there is either unmet demand or the potential for higher returns. The energy intensity of the industrial sector would drop if declining sectors used a lot of energy while growing sectors used significantly less. This means the country may keep or increase its industrial GDP even while it reduces its energy use; this might still be seen as an improvement in energy security.

The acceptability criterion looks at how new energy sources will affect people and the environment. A new energy technology must first get past political and environmental barriers to be widely used [49]. By switching to an energy mix that is based more heavily on renewable energy, a region’s energy security can be improved. Each type of renewable energy, such as nuclear, hydroelectric, solar, and wind power, has its own social, economic, and environmental concerns. It is measured by looking at two factors: the amount of CO2 released into the atmosphere and the amount of renewable energy used. Both embodied the concepts of acceptability and sustainability of energy sources.

Tongsopit et al. [49] described that having access to cheap energy resources is a key indicator of economic and social well-being along the affordability axis. Additionally, it addresses energy accessibility for all socioeconomic classes. If a country’s per-capita energy costs continue to rise, it will be increasingly difficult for it to meet the energy demands of its citizens. The natural logarithm’s calculation of the primary energy use per person and the proportion of renewable energy use in the total energy use are both indicators of affordability or development. Fang et al. [50] stated that being able to look at ways to improve energy security is a sign that the energy system has grown in the best, most eco-friendly, and low-carbon way possible. Table 1 describes the dimensions of energy security.

**Table 1:** Description of dimensions of energy security.

Availability	Definition	Unit	Description
Energy production to consumption Ratio (ES-AV1)	Ratio of the amount of primary energy produced to the amount of primary energy consumed	%	High value describes more availability of energy contributes to energy security positively.
Non-fossil energy (ES-AV2)	1- amount of fossil energy consumed to total energy consumed	%	More consumption of non-fossil energy means more availability of clean energy sources and more energy security
**Applicability**			
Energy Intensity (ES-AP)	Energy intensity level (Btu/2015$ GDP PPP)	Natural Logarithm	Low energy intensity describes more efficient production of goods and services and more energy security
**Acceptability/ Sustainability**			
Emission (ES-AC1)	Ratio of Carbon dioxide emission and gross domestic product	kg per 2015 PPP $ of GDP	Low emission from sources to GDP more acceptable and sustainable
Clean energy consumption (ES-AC2)	Amount of renewable energy consumed / total energy consumed	%	More consumption of renewable energy means more energy security in term of more consumption of clean energy sources
**Affordability/Develop-ability**			
Energy consumption per Capita (ES-AF)	Consumption of primary energy / total population	Natural logarithm	More per capita consumption of primary energy means more affordable energy and more developability in energy.

### 3.2. Independent variable

We used the most recent indicator that Wang et al. [51] recommended in their study. Researchers came up with the forward GVCs participation (fGVCP) and the backward GVCs participation (bGVCP) to measure how much a country is involved in global supply chains. The bGVCP, which also shows how much of the total value-added exports come from domestic intermediate exports, and the fGVCP, which shows how much of the value-added comes from domestic final goods and services, are both used to measure the value-added from GVCs. The first type is backward or downstream GVC participation. This is when a country adds foreign value to its own exports or uses imported materials to make exports. The latter is upstream GVC participation, where domestic value is added and then exported to a third country. A trading partner receives intermediate goods, reconditions them, and then exports them once more. Therefore, in this study, these two indices are added together to get the GVC’s participation degree for each country [51].


$$GVCP_{it}=fGVCP_{it}+bGVCP_{it}$$

Where fGVCP_it_ is equal to the ratio of domestic value added (DVA_it_) and gross export, while bGVCP_it_ is the ratio of foreign value added (FVA_it_) and gross export in country i at time t, the outcomes are in the range of 0 and 100; a value close to 100 depicts high participation. These GVC variables not only give us a more accurate picture of the GVC phenomenon, but they also help us fix the problem with the traditional approach, which undervalues the contribution of economies with a lower export orientation [52]. It was noted that developing nations’ GVC participation is lower than developed nations’ GVC participation.

### 3.3. Control variables and their impact on energy security

There are some control variables incorporated in this study. These are income level (IL), net inflows of foreign direct investment (NI-FDI), industrialization level (INdL), capital formation (CF), human capital index (HC), and political stability (PS). The income level of economies was measured as GDP per capita. For net inflows of FDI, we considered the net inflows as a percentage of GDP. The industrial sector’s contribution to GDP served as a proxy for the degree of industrialization of the various nations. The data for these independent variables can be found in the World Development Indicators (WDI) database. The data about the human capital index, which describes the country’s ability to organize its economic and professional potential, was obtained from Pen World Table.

The GDP per capita has a significant impact on the level of energy security. In general, higher GDP per capita is associated with higher energy consumption, as increased economic prosperity increases the demand for energy across all sectors of the economy [53]. A country’s ability to diversify its energy sources and invest in energy infrastructure can improve as its economy grows; however, this growth also increases the need to have access to consistent and secure energy sources [54, 55]. Therefore, countries with higher GDP per capita emphasize energy security initiatives, such as the development of local energy resources, investment in energy efficiency measures, and diversification of energy supply sources. However, countries with lower GDP per capita may be more susceptible to energy supply disruptions and price volatility because of the lack of capital to invest in energy infrastructure and security.

Energy security can be improved or jeopardized, depending on the extent of FDI from abroad. A country’s energy infrastructure can be strengthened and its dependency on a single energy source reduced if foreign direct investment (FDI) is attracted to the energy sector [56]. This has the potential to improve energy security by lowering the possibility of supply disruptions and price volatility. The inflow of FDI involves knowledge about new technologies and materials, production methods, or organizational management skills; hence, it is widely regarded as one of the key drivers of technology diffusion across borders. Osano and Koine [56] explained the impact of foreign direct investment (FDI) on growth in the economy by emphasizing the energy industry’s capacity-building, knowledge management, trade and export market access, skilled labor, and management and leadership development. Energy consumption and foreign direct investment (FDI) have been the subject of numerous studies, with mixed results. Two groups with different opinions exist about this connection. FDI cannot be explained by inefficient energy practices, and it is positively related to energy consumption. However, the degree to which FDI positively affects energy security depends on the regulatory environment of the host country and the extent to which FDI goals are aligned with national energy security objectives [57]. Energy security could be compromised by inappropriate or unregulated foreign direct investment (FDI), which could lead to overexploitation of natural resources, environmental damage, and inappropriate foreign control over domestic energy infrastructure. In order to take advantage of the benefits of FDI while protecting energy security concerns, it is crucial to ensure legal FDI practices and effective regulation.

The degree of industrialization has a significant effect on energy security. Owing to considerable manufacturing and production activities, highly industrialized countries often have a greater and more diverse energy demand. These countries are more vulnerable to disruptions in the supply of energy because this increase in demand often requires complex and resilient energy infrastructure. The dramatic increase in energy demand and consumption caused by industrialization has steadily increased the global energy consumption [58]. Increasing energy needs undermine energy security because of urbanization and industrialization’s concomitant rise in energy consumption. Jones [59] reported a positive correlation between energy security and industrialization, and the energy intensity elasticity of industrialization was 1.35. However, governments can engage in energy efficiency measures and the development of alternative and renewable energy sources because of technological advances and increased financial resources that accompany industrialization. Ma and Xu [60] argue that industrialization entails more than just an increase in the percentage of economic output attributable to industry. It also involves a process of structural transfer and updating caused by rising technological standards and new ideas. This approach has the potential to reduce energy intensity while simultaneously increasing the efficiency. Therefore, while industrialized nations may face greater energy security risks because of their reliance on energy-intensive industries, they also have resources to invest in strategies to improve their energy security, such as the adoption of renewable energy sources and the modernization of existing power plants. While less industrialized nations may have lower energy consumption, they may struggle to develop the infrastructure and resources needed to ensure that their energy supply is reliable.

The influence of human capital on energy security is substantial as it plays a crucial role in the development, management, and sustainability of energy systems. The presence of a highly educated and proficient labor force plays a crucial role in facilitating techno-logical advancements, up-holding vital energy infrastructure, devising efficient energy policies, and fostering the adoption of energy-efficient practices and conservation measures [61]. Insufficient human capital can result in vulnerabilities within energy supply chains, thereby impeding the dependability and adaptability of energy systems. Conversely, a competent and well-informed workforce has the potential to bolster energy security by diminishing reliance on unstable energy sources, mitigating environmental hazards, and guaranteeing a consistent and cost-effective energy supply that promotes economic and societal welfare. Theoretically, the influence of human capital on energy consumption can be observed through the effects of income and technology. In their study, Pablo-Romero and Sánchez-Braza [62] identified a noteworthy degree of substitutability between human capital and energy inputs. This finding implies that allocating resources towards the development of human capital may serve as a viable approach to achieving energy security objectives.

The reliability of energy supplies is heavily influenced by political stability. Political stability facilitates long-term planning, investment, and international cooperation, all of which are necessary for access to reliable and secure energy supplies. There is a lower likelihood of unexpected changes in policy that could disrupt energy supplies in politically stable countries because of the greater consistency with which energy policies are established and implemented [63]. In addition, political stability encourages both local and international investments in energy infrastructure [64], which in turn increases the reliability and diversity of energy sources. On the other hand, political instability may hinder energy production, distribution, and transportation as a result of conflicts, protests, or regime transitions [65, 66]. Geopolitical conflicts can develop when political unrest hinders international cooperation and diplomacy in securing energy supplies, posing a threat to energy security [67]. As a result, energy security relies heavily on political stability, which fosters the reliability, affordability, and sustainability of the energy supply. Table 2 presents the descriptive analysis of the variables.

**Table 2:** Descriptive analysis of variables.

	Average	SD	Minimum	Maximum
Developed Countries				
ES-AV1	0.67	0.32	0.05	1.89
ES-AV2	0.35	0.18	0.11	0.74
ES-AP	1.67	0.73	0.19	5.36
ES-AC1	0.43	0.31	0.07	3.02
ES-AC2	0.68	0.37	0.19	0.96
ES-AF	0.52	0.41	0.16	1.58
GVC	17.51	1.87	12.77	21.11
IL	10.03	0.74	8.22	11.37
NI-FDI	1.10	1.34	-7.20	5.63
CF	24.88	1.59	21.11	29.07
INDL	24.97	1.63	21.41	28.92
HC	3.13	0.34	2.07	3.85
PS	0.66	0.63	-1.63	1.76
Developing Countries				
ES-AV1	0.47	0.29	0.02	1.64
ES-AV2	0.21	0.14	0.09	0.57
ES-AP	1.88	0.61	0.11	6.74
ES-AC1	0.64	0.49	0.21	4.03
ES-AC2	0.42	0.35	0.13	0.73
ES-AF	0.46	0.23	0.09	1.12
GVC	14.86	2.17	11.06	20.68
IL	7.12	0.86	5.38	6.22
NI-FDI	0.79	0.75	-5.30	0.55
CF	22.17	2.06	19.32	21.04
INDL	22.31	2.09	19.49	20.53
HC	2.05	0.52	1.09	3.17
PS	14.08	1.75	11.06	18.26

### 3.4. Empirical model

The current study was designed to explore the dynamic relationship between GVC participation and energy security in developed and developing countries. The model used in this study can be described as follows:

$${ES}_{it}=\partial_{o}+\partial_{1}{LnGVC}_{it}+\partial_{2}{IL}_{it}+\partial_{3}{NF-FDI}_{it}+\partial_{4}{INDL}_{it}+\partial_{5}{CF}_{it}+\partial_{6}{HC}_{it}+\partial_{7}{PS}_{it}+\gamma_{t}+\eta_{i}+\mu_{ijt}$$

Here, ES describes the energy security of i^th^ economy in t time period. Similarly, ∂_i_ are parameters to be estimated while γ_t_ and η_i_ are incorporated in the model to explore the year and country fixed effect, and μ_ijt_ is the error term.

Before proceeding to the final analysis, we have checked the cross-sectional dependence at first from the empirical point of view [68]. Table 3 displays the outcomes of the cross-sectional Breusch-Pagan LM pre-estimation test. The results of four tests for cross-sectional dependency led to the rejection of the null hypothesis of cross-sectional independence. At the 1% level, it was discovered that all of the factors were statistically significant. Cross-sectional dependence between developed or developing countries is acknowledged and demonstrated, supporting the alternative view. That is, we shall proceed with the presumption that cross-sectional independence is disproved and that cross-sectional dependency is found for all variables.

**Table 3:** Cros-sectional dependency test’s results.

	Developed Countries				Developing Countries			
	Breusch-Pagan LM	Pesaran scaled LM	Bias-corrected scaled LM	Pesaran CD	Breusch-Pagan LM	Pesaran scaled LM	Bias-corrected scaled LM	Pesaran CD
ES-AV1	187.23*	18.33*	17.03*	10.23*	111.67*	23.12*	22.99*	12.32*
ES-AV2	54.87*	37.07*	36.89*	43.76*	37.23*	25.67*	25.098*	45.30*
ES-AP	13.21*	18.23*	17.99*	7.89*	10.36*	18.20*	17.67*	8.92*
ES-AC1	33.20*	22.99*	21.84*	29.34*	36.23*	31.43*	31.023*	19.23*
ES-AC2	62.34*	32.67*	32.01*	52.53*	54.44*	43.53*	43.001*	17.20*
ES-AF	10.98*	19.32*	17.23*	08.34*	13.28*	14.12*	13.67*	10.33*
lnGVC	178.92*	102.34*	99.78*	64.23*	155.25*	98.23*	98.112*	44.87*
IL	107.23*	98.23*	97.68*	45.77*	100.22*	87.94*	87.203*	33.09*
NI-FDI	59.56*	32.67*	31.79*	17.23*	41.55*	47.39*	46.77*	14.01*
INDL	83.78*	34.56*	33.29*	21.39*	49.37*	33.33*	33.102*	15.44*
CF	23.87*	29.32*	29.08*	16.32*	19.99*	43.89*	42.701*	12.22*
HC	1.45*	1.08*	1.05*	5.23*	1.55*	1.02*	1.005*	3.22*
PS	1.78*	1.69*	1.55*	6.22*	1.76*	1.34*	1.009*	4.59*

The data’s stationarity in the presence of CD has been tested using both the first- and second-generation unit root tests. The results of the LLC, IPF, FDF, and CIPS unit roots tests are shown in Table 4. The second-generation panel unit root tests allow for cross-sectional dependence, in contrast to first generation tests that assumed cross-section units were cross-sectionally independent. Different results regarding the stationarity of the variables described that only few variables were stationary at their level according to the both 1st and 2nd generation unit root tests. Many variables are initially nonstationary but become stationary after the first level of differentiation is applied.

**Table 4:** First generation and second-generation unit root test’s results.

Variables	Level/1st Difference	Developed Countries			Developing Countries		
		1st Generation		2nd Generation	1st Generation		2nd Generation
		LLC	FDF	CIPS (Pesaran 2007)	LLC	FDF	CIPS (Pesaran 2007)
ES-AV1	1(0)	-4.23	-8.12*	7.23*	-3.01	-5.11	8.03*
	1(I)	9.76*	17.23*	-15.33*	10.07*	16.10*	-18.02*
ES-AV2	1(0)	3.54	-6.23	5.02	1.54*	-3.53	4.98
	1(I)	-11.99*	14.67*	-20.3*	-09.99*	12.43*	-26.39*
ES-AP	1(0)	3.22	-7.23	-2.70	2.12	-4.93	-3.04
	1(I)	-11.44*	-16.22*	-14.09*	-09.43*	-15.02*	-12.50*
ES-AC1	1(0)	2.43	-3.33	5.30	1.23	-3.001	4.32
	1(I)	-13.8*	20.12*	-16.2*	-10.67*	18.83*	-23.25*
ES-AC2	1(0)	-1.07	-5.1	7.2	-2.43	-3.19*	5.32
	1(I)	-18.2*	21.23*	-13.44*	-14.09*	16.21*	-17.54*
ES-AF	1(0)	2.4*	-3.71	-3.01	1.87	-2.76	-3.99
	1(I)	-11.23*	16.23*	-8.20*	-09.13*	18.23*	-6.04*
lnGVC	1(0)	-10.22	-12.34*	-13.33	-16.02	-15.49	-10.05
	1(I)	-23.43*	24.33*	-32.22*	-28.22*	29.01*	-29.07*
IL	1(0)	10.98	-11.11	7.34	09.67	-13.66	6.54
	1(I)	-22.09*	25.21*	-20.03*	-32.10*	36.22*	-23.12*
NI-FDI	1(0)	10.23	-13.23	4.32	11.45	-09.28	2.22
	1(I)	-23.22*	25.35*	-19.21*	-17.76*	22.34*	-20.21*
INDL	1(0)	2.34	-3.45	1.08	1.54	-2.05	2.01
	1(I)	-20.33*	29.23*	27.21*	-19.99*	25.49*	28.61*
CF	1(0)	-3.2	-5.43	2.43	-1.2	-3.57	1.99
	1(I)	-33.21*	22.50*	-18.32*	-09.32*	10.02*	-11.32*
HC	1(0)	1.09	-1.88	2.1	0.981	-0.998	3.21
	1(I)	-8.65*	9.09*	-9.54*	-6.49*	7.20*	-10.55*
PS	1(0)	0.68	-0.71	1.01	1.03	-0.93	1.78
	1(I)	-2.54*	3.98*	-4.56	-3.44*	4.48*	-6.57*

*, **, and *** shows significance level at 1%, 5%, and 10% respectively.

Because CD is present and first-level difference data are stationary, we used the panel corrected standard error (PCSE) model in our study as suggested by Ha [69]. The coefficients provided by the PCSE are easily interpretable. Panel-corrected standard error (PCSE) models are specifically designed to address the issue of heteroscedasticity in the panel data. The utilization of PCSE models ensures reliable and effective parameter estimates in the presence of serial correlation or heteroscedasticity. The PCSE estimator is well acknowledged for its ability to address cross-sectional dependence (CSD) issues, and has expanded the range of solutions available for panel data estimation. The performance of the panel-corrected standard error (PCSE) estimation is greatly enhanced when the number of cross-sections exceeds the number of time periods (N > T). Estimators with panel-corrected standard errors (PCSE) address the issue of cross-sectional dependency in the dataset, thereby providing standard errors that are robust to both heteroscedasticity and autocorrelation. Furthermore, it has been found that the utilization of estimation methodologies incorporating panel corrected standard errors (PCSE) yields reliable estimation outcomes even when cross-sectional dependence is present [70]. We selected a feasible generalized least squares (FGLS) model for the additional robustness assessment because this method successfully addresses the potential endogeneity issue [71].

The study period of the current study is 1995–2018 because of the availability of the required data regarding each variable considered in the current study. Moreover, the current study considers both developed and developing countries because examining the impact of GVCs on the energy security systems of both developing and developed nations represents a strategic choice based on the urgent need to develop a full understanding of current global economic dynamics. The incorporation of both developing and developed nations permits a comparative examination that illuminates the complicated ways in which participation in global value chains affects energy security. Globally interconnected value chains (GVCs) have become crucial to the distribution and production of products and services, exerting a significant influence on energy consumption patterns and vulnerabilities in various countries. In light of this understanding, the use of a comparative method to analyze the international energy landscape allows for a comprehensive understanding, yielding significant insights that are of great value to policymakers and stakeholders in both established and emerging nations. We used the empirical method to clean up the data by leaving out countries with a lot of differences, missing observations, or outliers. This is important because both tests and the methods used need data that is very balanced. Following data cleansing, an empirical estimation is then performed using data from 72 nations including 35 developed and 27 developing nations.

We examine the cointegration of GVC and energy security in our data set in Table 5 using the Kao (KT), Westerlund (WT), and Pedroni (PT) cointegration tests. No cointegration is the null hypothesis for the Kao test, while all panels are cointegrated, which is the alternative hypothesis. No cointegration is the null hypothesis for the Pedroni test, and all panels are cointegrated, which is the alternative hypothesis. The null hypothesis for the Westerlund test is "no cointegration," and the alternative is "some panels are cointegrated." Cointegration has been confirmed at a 5% level of significance using the test. This means that the variables are I(1), and their long-term relationship has also been verified. In this study, the autoregressive distributed lag (ARDL) approach was employed to assess the short-term and long-term implications of GVC on energy security [72]. Therefore, dynamic fixed effects estimation in the ARDL model is required because of the possibility of endogeneity generated by the underlying link between GVC and energy security indicators, in addition to heteroscedasticity among countries [73]. It is important for the ARDL framework to have a dynamic fixed effects model because it deals with the problem of possible endogeneity that comes up because of the complicated relationship between GVC and energy security indicators. The inherent characteristics of GVC, characterized by their dynamic and mutual impacts on energy security, might give rise to endogeneity concerns. Furthermore, it is important to analyze the relevance of heteroscedasticity across countries, given the varying and evolving GVCs and energy security contexts. The dynamic fixed effects model is a good way to deal with the problems that were brought up. It gives us a way to understand the complicated link, lessen the effects of endogeneity, and take into account the presence of heteroscedasticity in the changing relationship between GVC and energy security over time.

**Table 5:** Kao, Pedroni and Westerlund cointegration tests.

	KT (DF)	WT (VR)	PT (PPt)
ES-AV1	-3.21	-1.99	-2.63
ES-AV2	-2.76	-2.32	-3.64
ES-AP	-4.92	-4.03	-2.67
ES-AC1	-1.78	-2.76	-2.10
ES-AC2	-2.64	-2.17	-2.64
ES-AF	-4.22	-1.77	

DF = Dickey-Fuller test, VR = Variance ratio, and PPt = Phillips-Perron t

### 3.5. Correlation matrix

Table 6 shows correlation scores among the variables incorporated in the study. All correlation values are less than 0.8, indicating that multi-collinearity may not be a problem for our model

**Table 6:** Correlation matrix.

**Developed Countries**													
	ES-AV1	ES-AV2	ES-AP	ES-AC1	ES-AC2	ES-AF	lnGVC	IL	NI-FDI	INDL	CF	HC	PS
ES-AV1	1												
ES-AV2	0.34	1											
ES-AP	0.43	0.33	1										
ES-AC1	-0.34	-0.23	-0.38	1									
ES-AC2	0.46	0.34	0.47	-0.43	1								
ES-AF	0.27	0.22	0.29	-0.12	0.54	1							
lnGVC	0.65	0.43	-0.54	-0.21	0.64	0.49	1						
IL	0.55	0.47	0.49	0.34	0.34	0.51	0.49	1					
NI-FDI	0.43	0.33	0.43	0.19	0.54	0.29	0.43	0.42	1				
INDL	-0.32	0.19	0.32	0.21	0.21	0.37	0.33	0.55	0.33	1			
CF	0.29	0.33	0.34	-0.29	0.17	0.31	0.19	0.47	0.29	0.3	1		
HC	0.47	0.52	0.54	-0.35	0.31	0.24	0.44	0.59	0.4	0.44	0.43	1	
PS	0.44	0.47	0.37	-0.29	0.22	0.3	0.59	0.64	0.48	0.61	0.51	0.39	1
**Developing Countries**													
	ES-AV1	ES-AV2	ES-AP	ES-AC1	ES-AC2	ES-AF	lnGVC	IL	NI-FDI	INDL	CF	HC	PS
ES-AV1	1												
ES-AV2	0.43	1											
ES-AP	0.39	0.39	1										
ES-AC1	-0.21	-0.37	-0.42	1									
ES-AC2	0.32	0.4	-0.41	-0.33	1								
ES-AF	0.19	0.29	0.34	-0.16	0.58	1							
lnGVC	0.41	0.51	-0.44	-0.33	0.42	0.23	1						
IL	0.33	0.32	0.41	0.42	0.38	0.43	0.47	1					
NI-FDI	0.27	0.28	0.49	0.17	0.49	0.23	0.45	0.34	1				
INDL	-0.39	0.16	0.36	0.23	0.33	0.41	0.41	0.52	0.29	1			
CF	0.31	0.22	0.3	-0.32	0.27	0.29	0.23	0.48	0.43	0.54	1		
HC	0.38	0.49	0.51	-0.38	0.34	0.32	0.33	0.42	0.36	0.39	0.45	1	
PS	0.54	0.42	0.35	-0.34	0.2	0.48	0.61	0.58	0.44	0.59	0.48	0.42	1

## 4. Results

### 4.1. Effect of GVCs on energy security of developed nations

This study looked at how global value chains affect the energy security of both rich and poor countries. Using PCSE estimates, we focus on how GVC affects energy security, as shown by the six variables that make up the four different aspects of energy security. The results for the developed countries show that their participation in the GVC had a significant effect on all energy security indicators. All of them were negatively impacted by GVC. It implies that the GVC participation of developed countries influenced the availability dimension negatively because it influenced the production of primary energy (ES-AV1) and non-fossil fuel consumption (ES-AV2) negatively. The GVC’s participation of developed countries does not harm acceptability in terms of its contribution to CO2 emissions. Using precise estimations of PCSE, Table 7 also illustrates the non-linear impact on energy security of GVC’s participation in developed nations in terms of its various indicators. The squared term of GVC significantly affected the indicators of energy security except CO2 emissions at a 1% level (ES-AC1). GVC has a positive impact on all indicators of energy security. Therefore, the ES-AV1, ES-AV2, ES-AP, ES-AC2, and ES-AF had a non-linear U-shaped relationship with GVC, while the ES-AC1 (CO2 emission) did not. It means that at first, the rise in GVC participation hurt the energy security systems of the developed countries, but that after a certain point, this rise in GVC participation may help the energy security systems of the developed countries.

**Table 7:** PCSE linear and non-linear effect of GVC on ES of developed countries.

**Linear effect of GVC on ES**						
Variables	ES-AV1	ES-AV2	ES-AP	ES-AC1	ES-AC2	ES-AF
lnGVC	-0.12* (0.001)	-0.17* (0.003)	-0.27* (0.009)	-0.018* (0.007)	0.11* (0.010)	-0.02* (0.004)
IL	0.27* (0.03)	0.023* (0.007)	-0.30* (0.07)	0.023* (0.009)	0.20** (0.08)	0.31* (0.002)
NI-FDI	0.13* (0.021)	0.022* (0.006)	-0.124* (0.0193)	-0.084* (0.021)	0.032** (0.011)	0.0032 (0.016)
INDL	-1.03* (0.19)	0.12* (0.08)	0.039* (0.002)	0.10* (0.007)	0.043** (0.017)	0.23** (0.089)
CF	0.001 (0.023)	-0.01 (0.90)	0.012 (0.05)	-0.021** (0.009)	0.006 (0.032)	0.038 (0.12)
HC	0.043* (0.003)	0.028 (0.021)	0.029* (0.002)	0.098* (0.013)	1.22* (0.067)	0.047 (0.052)
PS	0.56* (0.04)	0.03 (0.04)	0.15* (0.006)	-0.087* (0.014)	0.006** (0.002)	0.07 (0.06)
**Non-linear effect of GVCs on ES**						
lnGVC	-0.11* (0.002)	-0.15* (0.004)	-0.22* (0.007)	-0.019* (0.006)	-0.10* (0.009)	-0.03* (0.002)
LnGVC2	0.12* (0.006)	0.23* (0.019)	0.014* (0.002)	-0.027 (0.301)	0.09* (0.012)	0.19* (0.02)
IL	0.25* (0.04)	0.025* (0.004)	0.29* (0.061)	0.020* (0.007)	0.22** (0.07)	0.32* (0.008)
NI-FDI	0.11* (0.019)	0.02* (0.0049)	-0.12* (0.018)	0.08* (0.02)	0.029* (0.009)	0.003 (0.016)
INDL	-0.983* (0.17)	0.10* (0.06)	0.035* (0.006)	0.14* (0.007)	0.041** (0.014)	0.20** (0.069)
CF	0.002 (0.024)	-0.013 (0.91)	0.010 (0.06)	-0.020** (0.009)	0.005 (0.030)	0.037 (0.10)
HC	0.040* (0.005)	0.026 (0.023)	0.027* (0.002)	0.097* (0.012)	1.20* (0.064)	0.045 (0.050)
PS	0.55* (0.03)	0.04 (0.039)	0.14* (0.005)	-0.086* (0.013)	0.007* (0.001)	0.06 (0.05)

*, and ** shows statistical difference at 1% and 5% respectively.

There is a positive correlation between higher income levels and many energy security-related indicators. Specifically, an increase in income level is associated with considerable increases in primary energy production (ES-AV1: 0.27), non-fossil fuel consumption (ES-AV2: 0.023), the ratio of carbon dioxide emissions to GDP (ES-AC1: 0.023), clean energy consumption (ES-AC2: 0.20), and energy consumption per capita (ES-AF: 0.31). NI-FDI positively affects primary energy production (ES-AV1: 0.13), non-fossil fuel consumption (ES-AV2: 0.022), clean energy consumption (ES-AC2: 0.032), and negatively affects energy intensity (ES-AP: -0.124) and the ratio of carbon dioxide emissions to GDP (ES-AC1: -0.084). Industrialization negatively affects primary energy production (ES-AV1: -1.03) and positively affects non-fossil fuel consumption (ES-AV2: 0.12), energy intensity (ES-AP: 0.039), the ratio of carbon dioxide emissions to GDP (ES-AC1: 0.10), clean energy consumption (ES-AC2: 0.043), and energy consumption per capita (ES-AV1: 0.23). Capital formation (CF) only has a significant effect on ES-AC1 (primary energy production), with a coefficient value equal to -0.021 at p < 0.05. Human capital (HC) is positively and significantly associated with ES-AV1, ES-AP, ES-AC1, and ES-AC2. The impact of political stability (PS) on ES-AV1, ES-AP, and ES-AC2 is significant and positive, while on ES-AC1 it is negative.

### 4.2. Effect of GVCs on energy security of developing nations

The PCSE estimate shows that there is statistically significant evidence that GVC affects indicators of energy security. Notably, GVC has a negative effect on the ratio of energy production to energy consumption (ES-AV1), the use of non-fossil fuels (ES-AV2), and the use of renewable energy per person (ES-AF). The remaining indicators are also positively impacted at the same time at the 1% significance level. The results imply that the initial improvement of GVC compromises the security of the developing countries’ energy systems. This result also indicates that the energy security of developing countries suffers in terms of availability, applicability, acceptability, and affordability as the value of GVC rises. Participation in GVC by developing countries results in higher energy consumption and pollution emissions but a decrease in energy production. If these nations accept membership in GVC, their energy systems are more likely to become vulnerable. In Table 8, the square term of GVC was also added, and a significant impact on energy security indicators was found. The PCSF estimates for the square term of GVC show that it has negative and statistically significant effects on ES-AP, ES-AC1, and ES-AC2 in particular. In comparison, GVC has statistically significant and positive effects on ES-AV1, ES-AV2, and ES-AF. The non-linear interactions between the ES-AV1, ES-AV2, and ES-AF were specifically U-shaped, while those between the ES-AP, ES-AC1, and ES-AC2 were inverted-U shapes. It means that at first, energy use and CO2 emissions go up in developing countries as their participation in GVC grows, but that after a certain point, both start to go down as GVC participation grows. Also, as the participation of developing countries in GVC grows, the use of non-fossil fuels first goes down and then goes up.

**Table 8:** PCSE linear and non-linear effect of GVC on ES of developing countries.

**Linear effect of GVC on ES**						
Variables	ES-AV1	ES-AV2	ES-AP	ES-AC1	ES-AC2	ES-AF
lnGVC	-0.09* (0.003)	-0.14* (0.017)	0.15* (0.019)	0.021* (0.008)	0.17* (0.014)	-0.11* (0.012)
IL	0.19* (0.037)	0.10* (0.012)	0.09* (0.016)	0.003** (0.001)	0.11** (0.02)	0.26* (0.032)
NI-FDI	0.073* (0.012)	0.031** (0.011)	-0.043 (0.117)	-0.054* (0.017)	0.016** (0.007)	0.027 (0.016)
INDL	-0.79* (0.13)	0.04* (0.005)	0.022* (0.006)	0.01* (0.003)	0.013** (0.011)	0.32** (0.075)
CF	0.02* (0.006)	0.002 (0.15)	0.044 (0.02)	-0.34* (0.029)	0.001 (0.072)	0.030 (0.17)
HC	0.143* (0.023)	0.06** (0.021)	0.06* (0.002)	0.132* (0.014)	0.99* (0.067)	0.243 (0.03)
PS	0.44** (0.12)	0.08 (0.12)	0.09* (0.003)	-0.071** (0.020)	0.012** (0.005)	0.13 (0.02)
**Non-linear effect of GVCs on ES**						
lnGVC	-0.08* (0.002)	-0.13* (0.016)	0.16* (0.018)	0.022* (0.007)	0.16* (0.013)	-0.10* (0.011)
LnGVC2	0.23* (0.009)	0.30* (0.019)	-0.009** (0.003)	-0.019** (0.006)	-0.07* (0.011)	0.28* (0.03)
IL	0.18* (0.036)	0.11* (0.011)	0.08* (0.015)	0.002** (0.001)	0.10** (0.01)	0.25* (0.031)
NI-FDI	0.070 (0.010)	0.029 (0.012)	-0.039 (0.115)	-0.053 (0.016)	0.015 (0.007)	0.026 (0.014)
INDL	-0.78* (0.12)	0.03* (0.004)	0.021* (0.005)	0.011* (0.002)	0.014** (0.012)	0.31** (0.074)
CF	0.021* (0.005)	0.0018 (0.14)	0.043 (0.03)	-0.35* (0.028)	0.0011 (0.070)	0.031 (0.16)
HC	0.142* (0.022)	0.05** (0.020)	0.05* (0.001)	0.131* (0.013)	0.98* (0.066)	0.244 (0.04)
PS	0.43** (0.119)	0.076 (0.117)	0.08* (0.002)	-0.072** (0.019)	0.013** (0.006)	0.12 (0.01)

*, and ** shows statistical difference at 1% and 5% respectively.

There is a positive correlation between higher income levels and many indicators of energy security. In particular, an increase in income is linked to a large increase in primary energy production (ES-AV1:0.19), non-fossil fuel consumption (ES-AV2:0.10), energy intensity (ES-AP:0.0.09), ratio of carbon dioxide emissions to GDP (ES-AC1:0.003), clean energy consumption (ES-AC2:0.11), and energy consumption per capita (ES-AF:0.26). NI-FDI positively affects primary energy production (ES-AV1: 0.073), non-fossil fuel consumption (ES-AV2: 0.031), clean energy consumption (ES-AC2: 0.016), and negatively affects the ratio of carbon dioxide emissions to GDP (ES-AC1: -0.05). Industrialization negatively affects primary energy production (ES-AV1: -0.79) and positively affects non-fossil fuel consumption (ES-AV2: 0.04), energy intensity (ES-AP: 0.022), the ratio of carbon dioxide emissions to GDP (ES-AC1: 0.01), clean energy consumption (ES-AC2: 0.013), and energy consumption per capita (ES-AV1: 0.32). Capital formation (CF) only has a significant effect on ES-AC1 (primary energy production), with a coefficient value of 0.02, and a significant negative impact on ES-AC1 (-0.34) at p < 0.05. Human capital (HC) is positively and significantly associated with ES-AV1, ES-AV2, ES-AP, ES-AC1, and ES-AC2. The impact of political stability (PS) on ES-AV1, ES-AP, and ES-AC2 is significant and positive, while that on ES-AC1 is negative.

### 4.3. Robustness check

The FGLS estimates in Table 9 were quite similar to PCSE. Both types of estimates (PCSE and FGLS) confirm the existence of a non-linear relationship between GVC and the energy security systems of developed and developing countries.

**Table 9:** FGLS linear and non-linear effect of GVC on ES.

Developed Countries						
Linear effect of GVC on ES						
Variables	ES-AV1	ES-AV2	ES-AP	ES-AC1	ES-AC2	ES-AF
lnGVC	-0.09* (0.002)	-0.14* (0.016)	0.15* (0.018)	0.021* (0.007)	-0.17* (0.015)	-0.11* (0.011)
Control Variables	Yes	Yes	Yes	Yes	Yes	Yes
Non-linear effect of GVCs on ES						
lnGVC	-0.08* (0.001)	-0.13* (0.017)	0.16* (0.017)	0.022* (0.006)	-0.16* (0.012)	-0.10* (0.010)
LnGVC2	0.23* (0.008)	0.23* (0.018)	-0.009** (0.002)	-0.019** (0.005)	0.07* (0.010)	0.18* (0.02)
Control Variables	Yes	Yes	Yes	Yes	Yes	Yes
Developing Countries						
Linear effect of GVC on ES						
Variables	ES-AV1	ES-AV2	ES-AP	ES-AC1	ES-AC2	ES-AF
lnGVC	-0.09* (0.005)	-0.14* (0.019)	0.15* (0.020)	0.021* (0.009)	-0.17* (0.017)	-0.11* (0.014)
Control Variables	Yes	Yes	Yes	Yes	Yes	Yes
Non-linear effect of GVCs on ES						
lnGVC	-0.08* (0.003)	-0.13* (0.017)	0.16* (0.020)	0.022* (0.010)	-0.16* (0.018)	-0.10* (0.016)
LnGVC2	0.23* (0.023)	0.30* (0.034)	-0.009** (0.008)	-0.019** (0.010)	0.07* (0.013)	0.28* (0.047)
Control Variables	Yes	Yes	Yes	Yes	Yes	Yes

*, and ** shows statistical difference at 1% and 5% respectively.

### 4.4. GVC’s short- and long-term impact on developed and developing nations’ energy security

We find that in the short run, GVC only has a statistically significant effect on ES-AV1, ES-AP, ES-AC1, and ES-AC2, but not on ES-AF, which is about the developed countries. It is noteworthy that GVC participation had beneficial effects on ES-AC2 while having positive effects on ES-AP and ES-AC1 in the short run (Table 10). It implies that the developed countries participation in GVC had a drastic impact on energy security in the short run. Furthermore, for each of the six measures of the long-term impact, GVC is statistically significant at 1% or 5%. GVC has a detrimental effect on ES-AV1. GVC, on the other hand, has a positive effect on ES-AV2, ES-AP, ES-AC1, ES-AC2, and ES-AF. The most striking effects are on ES-AV2, ES-AC1, and ES-AC2. It implies that in the long run, the participation of developed countries in GVC may generate a positive impact on their energy security system in terms of high consumption of non-fossil energy, low intensity of energy, low emission of carbon dioxide, and high consumption of renewable energy. In the long run, the participation of developed countries in GVC can increase the availability, applicability, acceptability/sustainability, and affordability of energy security.

**Table 10:** Long run and short run effects of GVC and energy security system of developed and developing countries.

Variables	ES-AV1	ES-AV2	ES-AP	ES-AC1	ES-AC2	ES-AF
Developed countries						
SR effect						
D. LnGVC	-0.004** (0.0009)	0.021 (0.026)	0.019* (0.003)	0.009* (0.0016)	0.026* (0.003)	0.01 (0.032)
Control Variables	Yes	Yes	Yes	Yes	Yes	Yes
LR effect						
lnGVC	-0.012** (0.004)	0.31* (0.013)	-0.18* (0.02)	-0.36* (0.043)	0.25* (0.046)	0.10** (0.042)
Control Variables	Yes	Yes	Yes	Yes	Yes	Yes
Developing countries						
D. LnGVC	-0.215* (0.009)	-0.009** (0.002)	0.003 (0.027)	-0.016 (0.025)	0.043 (0.503)	0.0001 (0.032)
Control Variables	Yes	Yes	Yes	Yes	Yes	Yes
LR effect						
lnGVC	-0.036** (0.012)	0.093* (0.017)	0.301* (0.031)	0.44* (0.045)	0.18* (0.032)	0.093** (0.038)
Control Variables	Yes	Yes	Yes	Yes	Yes	Yes

LR = long run, and SR = short run

In the case of developing countries, we discover that GVC only affected ES-AV1 and ES-AV2 significantly in the short run. It is important to note that GVC’s involvement had significant short-term negative effects on the amount of energy available in developing countries. It implies that the developing countries participation in GVC may increase their consumption of primary energy and lower their non-fossil energy consumption in the short run. Moreover, for each of the six measures of the long-term impact, GVC has a positive and statistically significant effect on all energy security indicators except ES-AV1. Therefore, the long-term participation of developing countries in GVC may have a favorable effect on energy security. The most striking effects are on ES-AC1 and ES-AP. It implies that in the long run, if developing countries join GVC, it could lead to a rise in the amount of non-fossil, renewable, and primary energy used per person, as well as a drop in CO2 emissions and energy intensity. Long-term trends show that nations with larger scales within the GVC or higher GVC participation consume more energy overall but release less CO2, which favors the use of non-fossil fuel energy sources.

## 5. Discussion

By focusing on different parts of energy security, such as acceptability, applicability, acceptability/sustainability, and affordability/developability, the study results specify a fresh logical viewpoint on the factors that contribute to environmental deterioration and an inclusive analysis of the energy security frameworks in developed and developing nations. It was seen that GVC had a huge effect on the energy security systems of both developed and developing nations. GVC partners may think that environmental value chain governance implementations aren’t good enough, which could lead to the conclusion that GVC makes the energy system vulnerable [27]. Additionally, when there are sufficient and effective penalties in place, smaller and less competitive GVC members are more likely to adhere to environmental sustainability. The other main point is that it is hard for GVC partners to implement and measure environmental aspects of sustainability because it is hard for institutions to share green technologies [29]. The study findings underline how critical GVC membership is for protecting the energy grid [43]. Through the transfer of knowledge and technology, GVC’s participation increases the amount of knowledge, skills, innovation, and technology that can be used.

The results of this study show that as the value of GVC goes up, the energy systems of developing countries become less available, applicable, acceptable, sustainable, affordable, and easy to build. When developing countries join GVC, their energy use, energy intensity, and pollution emissions all go up, but their energy output goes down. If these countries join GVC, there is a greater chance that their energy systems will become vulnerable. Here are several plausible justifications for why this is the case in developing nations. First, the economies of developing nations tend to be categorized by marginal production; this is because the transition from resource-based sectors and labor-intensive to heavy industries is directly linked to the process of shifting from marginal production to the core of the worldwide trade system of a diversified and environmentally harmful product mix. Consequently, due to the multiplier effect of industrialization and more manufacturing, energy security will become more and more at risk as the global trade network grows. However, there is a chance that the gains in money and economic expertise may not lead to major improvements in high-tech breakthroughs and environmental concerns among individuals and firms unless there is a far higher degree of engagement in the global trade network [71, 74]. In contrast to the use of non-renewable energy, emerging or low-income countries use of renewable energy technology is less common [75] because of high start-up costs and a lack of environmental concerns.

In fact, participation in GVC may force developing nations to adopt cutting-edge technology that is beyond their capabilities to do so [76]. In order to compete in global markets, producers must also adhere to high standards that are impossible for them to meet with their current technology and labor force [77]. Last but not least, participation in GVC enhances the effectiveness of private rules and governmental institutions, which are thought to be important means of achieving environmental sustainability. However, weak and subpar institutional systems are frequently a feature of low-income nations [78]. As a result, there may be negative effects on the energy security of these nations.

The internationalization of GVC’s intra-product activity has a significant impact on emerging nations, especially China. On the one hand, GVC goods grew more technologically advanced and complex with the passage of time. The spread of management knowledge and technology could aid original equipment manufacturers in emerging nations and improve energy and environmental efficiency [79, 80]. However, taking part in GVC may increase energy use and CO2 emissions because developing nations often produce the majority of the world’s energy- and carbon-intensive goods, such as electrolytic aluminum. In this respect, taking part in GVC could have a negative impact on energy conservation and pollution prevention, which are results that are typically referred to as pollution refuge. By outsourcing the low value-added linkages to developing nations, developed countries are able to maintain their position as high value-added links (such as R&D) in domestic industry. These connections are typically carbon- and energy intensive [81, 82].

Developed nations are in the negative for pollution emissions in global trade, importing directly or indirectly from developing nations goods that require a lot of pollution to produce [23]. So, GVC makes it possible for potentially harmful substances and pollution-causing agents to move from developed to developing countries. Because of the significant differences between developed and developing countries in GVC involvement and carbon intensity, emerging countries face environmental risks, such as a rise in CO2 intensity. When developed countries take part in the GVC, their carbon intensity goes down a lot, but when developing countries take part, their carbon intensity goes up [81]. A study described how participation in GVC increases the amount of energy used and CO2 emissions and also makes the environment worse [82]. They worry that the large differences between developed and developing countries in technology and industrial structure will also have a big effect on the amount of GVC involvement and carbon intensity [30, 83]. For instance, developed nations have an advantage over emerging nations in terms of capital and technology, occupying the GVC of numerous industries upstream. These links of high value addition, such as product development and product design in the GVC, are associated with low carbon and energy intensity [84]. On the other hand, because developing nations process and assemble parts in a labor- and energy-intensive manner, they remain at the downstream end of the GVC, which precludes them from receiving any potential benefits [10]. With their technological advantages, rich countries transfer carbon-intensive manufacturing facilities to developing nations through the GVCs division scheme. Therefore, under production-based accounts, emerging countries often dominate the world’s carbon-intensive industries, which results in an increase in energy consumption and carbon emissions [79]. A different school of thought contends that technological spillovers and labor transfers inside GVCs help to spread environmental and new energy technologies, lower emissions, and promote the usage of renewable energy sources [85].

It is important to remember that only less competitive and small-scale GVC participants face difficulties in obtaining the environmentally friendly GVC. As we previously suggested, after a certain point in the GVC’s expansion, GVC membership’s negative consequences on energy security become positive. Previous research has shown that there is a link between a country’s knowledge, understanding, and carbon dioxide emissions and its economic complexity and product proximity that is negative [69] and a link that is positive [50, 86] between these measures and the ability to develop and afford energy security. It is crucial to keep in mind that a nation that is a part of the GVC network aids other nations in acquiring new knowledge and skills through a process of information and technology transfer [44, 87], which affects their energy security. This is brought up in Le et al.’s [71] discussion of the non-linear effects of economic diversification on the many parts of energy security. The improvement of GVC values increases the energy system’s accessibility, acceptability, developability, and sustainability. However, as we previously argued, the benefits of GVC participation only materialize if they exceed a particular threshold. Otherwise, GVC harms nations’ ability to expand their economies sustainably, regardless of their level of income. Our results are in line with other research [88], that emphasizes the importance of economic complexity in explaining potential relationships between primary energy consumption, emissions of CO2, and energy intensity. Pollution control and ISO 14001 environmental certification are two examples of how GVC participation helps a country develop its own advantages to fight environmental degradation [36]. With their technological advantages, developed countries transfer carbon-intensive manufacturing facilities to developing nations through the GVCs division scheme. Therefore, under production-based accounts, developing countries often dominate the world’s carbon-intensive industries, which results in an increase in energy consumption and carbon emissions [79]. A different school of thought contends that technological spillovers and labor transfers inside GVCs help to spread environmental and new energy technologies, lower emissions, and promote the usage of renewable energy sources.

There is a favorable relationship between income level and overall energy security indicators, except for energy intensity and emissions in developing countries and only emissions in developed countries. Generally, countries characterized by a greater GDP per capita place significant emphasis on energy security through the implementation of diversified energy sources, substantial investments in infrastructure, and the adoption of efficiency measures [54, 55]. Based on the results regarding NI-FDI and energy security, shed light on the importance of FDI in order to improve energy security. Foreign direct investment (FDI) has the potential to bolster energy security through the attraction of money and technological breakthroughs [56, 89–91]. However, the extent of its influence is contingent upon the alignment of regulatory frameworks. The process of capital formation has a critical role in enhancing the resilience of energy infrastructure and mitigating susceptibility. Industrialization has a varied impact on individual indicators of energy security, but in general, industrialized nations encounter heightened demand but possess the capacity to allocate resources towards the implementation of energy security plans [59]. The influence of human capital on energy consumption is manifested through the factors of income and technology, hence exerting an impact on the attainment of energy security objectives [13, 14, 92, 93]. The promotion of political stability is essential for fostering long-term planning, investment, and international cooperation, all of which are critical factors in ensuring the reliability and security of the energy supply.

## 6. Conclusions

This study is the first to look at how GVC affects both developing and developed countries’ energy security systems. Using data from a sample of 35 developed countries and 27 developing countries around the world from 1995 to 2018, we draw important conclusions. The study confirmed the rationality of the GVC Partnership’s non-linear relationship with primary energy consumption, non-fossil energy use (items of the availability dimension), energy intensity (applicability), renewable energy use (acceptability/sustainability), and primary energy consumption per capita (affordability) with regard to developed countries. This means that the GVC’s negative impact on some parts of energy security will change for the better if the level of development in GVC countries reaches a certain point. Moreover, we could not find a significant non-linear relationship between GVC and CO2 emissions for developed countries. It implies that the developed countries participation will increase and CO2 emissions will decrease, and no turning point exists herein. We discovered a non-linear relationship between developing nations’ participation and their indicators of energy security. This implies that the participation of developing countries in GVC will first increase CO2 emissions and energy intensity and, after reaching a certain point, participation in GVC will reduce emissions and energy intensity. In other words, if developing nations’ participation in GVC reaches a particular level and its negative effects on all facets of energy security turn out to be favorable, it may produce favorable results. Additionally, there is proof of long-term links between GVC participation and the energy security of developed and developing countries, and our results support their long-term ramifications.

Income increases positively affect the energy security indices in both developed and developing economies. Income growth is associated with considerable increases in primary energy output, non-fossil fuel consumption, the carbon dioxide emissions-to-GDP ratio, clean energy use, and per capita energy consumption. In developed countries, industrialization hurts the primary energy output. It has been found to positively affect non-fossil fuel consumption, energy intensity, the carbon dioxide emissions-to-GDP ratio, clean energy consumption, and energy consumption per capita. Research has shown that capital formation (CF) hurts primary energy production. High human capital (HC) and political stability (PS) correlate positively and significantly with energy security indices. Industrialization in emerging countries has similar negative effects on primary energy output. It also boosts non-fossil fuel consumption, energy intensity, carbon dioxide emissions to GDP ratio, clean energy consumption, and energy consumption per capita. Capital formation reduces primary energy output but increases carbon dioxide emissions and GDP. Except for the carbon dioxide emissions to GDP ratio, human capital (HC) and political stability (PS) correlate positively and statistically with most energy security indices.

Therefore, developing countries have to update their technologies and use less energy to compete on the global market. They should work to improve technology, which can increase energy efficiency while lowering energy intensity and the damage that industrial processes can do to the environment by making it dirty. Therefore, it’s possible that greater trade won’t have a big influence on pollution in wealthy nations. Representatives from developed nations should use a variety of strategies to pique the interest of their potential business partners in sustainable technologies and products. These strategies may include spreading the word about the developed nations ‘cutting-edge innovations, discussing the challenges and potential solutions for achieving efficiency in energy use, and sharing the developed nations ‘experience with their prospective partners and the financial benefits of modern energy management practices. The government should encourage companies to use technology more widely in order to scale back emissions of greenhouse gases and boost energy-use efficiency. Particularly, firms should adopt cutting-edge technology to lessen the primary energy intensity, hence boosting sustainable production energy or lowering the intensity of energy consumption. Participating in GVC is one of the greatest ways to get this kind of contemporary technology.

Policymakers must prioritize comprehensive and diversified plans to improve energy security. First, we should establish probusiness policies and invest in human capital in order to provide revenue growth prospects. Income levels correlate positively with the energy security indicators, making this significant. Balanced industrialization must be promoted with a focus on renewable energy and energy efficiency. Promoting renewable and low-carbon energy sources is crucial, especially because income levels are positively correlated with clean energy use. Political stability is important, because disruptions can threaten energy security. The negative effects of income on carbon emissions must be addressed by reducing emissions and boosting energy efficiency. These guidelines are supported by continuous monitoring, worldwide cooperation, and energy efficiency. These elements can help build reliable and sustainable energy systems in developed and developing countries.

The results of this study signify the complex relationship between global value chains (GVC), income, industrialization, and several energy security measures in developed and developing countries. However, it is important to recognize certain potential limits. The selected sample size, consisting of 35 developed and 27 developing countries, provided interesting insights. However, it is important to acknowledge that this sample may not comprehensively represent the diverse range of energy security dynamics in the global context. This constraint underscores the need for prudence when generalizing the findings to a wider global scope. It is worth noting that this study relied heavily on data collected between 1995 and 2018. Although the aforementioned timeline provides insightful historical perspectives, it is crucial to recognize that energy security is a field that is constantly changing owing to advancements in technology, adjustments in policy settings, and changes in geopolitical dynamics. Therefore, it is possible that this study does not comprehensively encompass current advancements that could have a substantial impact on energy security outcomes.

## Supporting information

S1 Data (XLSX)
